# Cadmium Transport in Maize Root Segments Using a Classical Physiological Approach: Evidence of Influx Largely Exceeding Efflux in Subapical Regions

**DOI:** 10.3390/plants12050992

**Published:** 2023-02-21

**Authors:** Alberto Rivetta, Michele Pesenti, Gian Attilio Sacchi, Fabio Francesco Nocito, Maurizio Cocucci

**Affiliations:** Dipartimento di Scienze Agrarie e Ambientali—Produzione, Territorio, Agroenergia (DiSAA), Università degli Studi di Milano, 20133 Milano, Italy

**Keywords:** *Zea mays* L., root, cadmium, calcium, transport

## Abstract

The bidirectional fluxes of cadmium and calcium across the plasma membrane were assessed and compared in subapical maize root segments. This homogeneous material provides a simplified system for investigating ion fluxes in whole organs. The kinetic profile of cadmium influx was characterized by a combination of a saturable rectangular hyperbola (*K_m_* = 30.15) and a straight line (*k* = 0.0013 L h^−1^ g^−1^ fresh weight), indicating the presence of multiple transport systems. In contrast, the influx of calcium was described by a simple Michaelis–Menten function (*K_m_* = 26.57 µM). The addition of calcium to the medium reduced cadmium influx into the root segments, suggesting a competition between the two ions for the same transport system(s). The efflux of calcium from the root segments was found to be significantly higher than that of cadmium, which was extremely low under the experimental conditions used. This was further confirmed by comparing cadmium and calcium fluxes across the plasma membrane of inside-out vesicles purified from maize root cortical cells. The inability of the root cortical cells to extrude cadmium may have driven the evolution of metal chelators for detoxifying intracellular cadmium ions.

## 1. Introduction

Cadmium (Cd) is one of the most toxic heavy metals naturally present in the soil and it is largely released into the environment by anthropic activities [1,2,3]. Although not essential for plant nutrition, Cd^2+^ is rapidly taken up by plants and accumulated in vegetative and reproductive organs, which may represent the main entrance points of cadmium into the food chain [4,5].

The free hydrated ionic form of cadmium (Cd^2+^) present in the soil solutions is considered the main source of the element available for plant uptake [6]. Part of these Cd ions adsorbs on the negative charges of the cell wall surrounding the plasma membrane of the root cells. Others diffuse throughout the root apoplast until they move across the plasma membrane using integral membrane proteins [7].

Since Cd does not play any role in the cells, no specific transporters for this element have evolved in plants. It is generally assumed that Cd^2+^ uptake in plants occurs through plasma membrane transporters involved in the absorption of mineral nutrients, such as calcium, iron, zinc and manganese, which share with Cd^2+^ similar chemical characteristics [7,8]. Several works indicate the involvement of IRT1 (IRON-REGULATED TRANSPORTER-1)—a metal transporter with a broad substrate range—and other members of the zinc-regulated, iron-regulated transporter-like protein (ZIP-IRT) family in Cd^2+^ uptake [9,10,11,12,13]. Other works demonstrated that members of the NRAMP (natural resistance-associated macrophage protein) family, generally involved in the absorption of Fe and Mn ions from the soil, are the major pathways for Cd^2+^ uptake in rice [14,15]. The significance of these transport systems in mediating Cd^2+^ uptake is strongly dependent on plant nutritional status that in turn modulates the expression of each transporter on the plasma membrane of the root cells [14,15,16].

In animal cells, calcium (Ca^2+^) channels are implicated in cadmium uptake [17,18,19], since divalent Cd^2+^ and Ca^2+^ have very similar physicochemical properties [20,21]. In plants, several observations confirm the involvement of Ca^2+^ transport proteins in mediating Cd^2+^ uptake. In germinating radish seeds, Ca^2+^ reduced Cd^2+^ uptake and toxicity [22]. Other papers have revealed that Ca^2+^ and Cd^2+^ are antagonistic ions competing for uptake when they co-occur in the incubation media, suggesting the involvement of the Ca^2+^ channel in Cd^2+^ uptake [23,24,25].

Once inside the cells, the excess of Cd ions is mainly detoxified through specific mechanisms based on chelation and subcellular compartmentalization that allow cells to retain Cd^2+^ into the vacuoles [8]. Phytochelatins have largely been described as the main class of Cd^2+^ chelators involved in these processes [26,27], although different transporters have been described as directly involved in moving free Cd ions across the tonoplast [28,29,30,31]. Cd ions escaping these trap systems are potentially available to be exported outside the symplast [32]. Although Cd-resistant bacteria can directly extrude Cd ions from the cytosol through metal ATPases [33], in plants, similar transport activities—mediated by HMA P-Type ATPases—have been largely described as involved in controlling xylem loading in the root stele and then root-to-shoot Cd^2+^ translocation [32,34,35]. However, the existence of a root Cd^2+^ efflux was hypothesized by Costa and Morel [36] as a possible detoxification mechanism limiting Cd accumulation in the root cells of *Lupinus albus*. No direct evidence concerning the involvement of Ca^2+^ transporters in Cd^2+^ efflux has been reported in plants.

Notwithstanding the existence of a lot of information about the transporters potentially involved in mediating Cd^2+^ fluxes across the plasma membrane of the root cells, little is known about how their activities result in Cd^2+^ accumulation in a whole organ. Here we present a detailed characterization of both Cd^2+^ and Ca^2+^ bidirectional fluxes across the plasma membrane to provide a glimpse into the integration of different transport systems in a whole plant organ. To do this we used a classical physiological approach on non-Cd^2+^ stressed subapical maize root segments. Differences between Cd^2+^ and Ca^2+^ fluxes are also discussed in relation to the main mechanisms involved in Cd^2+^ detoxification.

## 2. Results

### 2.1. Short Term Cd^2+^ Influx in Maize Root Segments

Most of the experiments presented in this work were conducted using subapical maize root segments (hereafter also called “root segments”), cut between 2 and 8 mm from the tips of the main roots. This portion of root shows a differentiation which mainly consists of parenchyma tissue and epidermis, with only some protoxylem vessels already defined near the cut at 8 mm from the tip. The extremely simplified and rather homogeneous structure of the segments is thus suitable for studying ion fluxes in a whole organ, avoiding the complexity of the whole root. The choice of this material to study cadmium cell fluxes is consistent with studies on the spatial profile of Cd^2+^ uptake along the root axe, where the highest Cd^2+^ influx is sustained by the apical region of the root in wheat and rice [37,38].

Cadmium toxicity to a variety of cell types is abundantly described in the literature [39,40,41,42,43,44,45,46] and essentially depends on concentrations and time of exposure. Thus, we checked whether cadmium could induce toxicity in our experimental conditions. Relatively short-term incubations (up to 45 min) of root segments in media containing a wide range of Cd^2+^ concentrations (up to 500 µM) did not affect respiration (rate of O_2_ uptake) or cell membrane integrity measured as potassium leakage in the medium, suggesting that, at least in our conditions, Cd^2+^ exposure did not produce generalized toxic effects.

**Figure 1:**
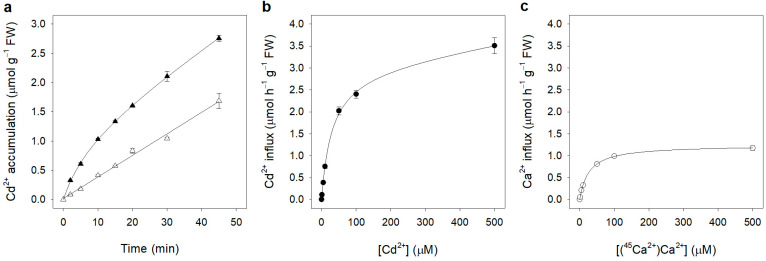
(**a**) Time-course of Cd^2+^ accumulation in maize root segments. Root segments were incubated in the presence of 50 µM CdCl_2_ for 2, 5, 10, 15, 20, 30 and 45 min. At the end of the incubation, root segments were washed twice in water (filled triangles) or in 1 mM LaCl_3_ (open triangles). The amount of spurious Cd^2+^ present in the root segments before incubation was negligible (0.18 ± 0.04 nmol g^−1^ FW). (**b**) Kinetic of Cd^2+^ influx in maize root segments. Root segments were incubated for 15 min in the presence of 1, 5, 10, 50, 100 and 500 μM CdCl_2_, harvested, washed twice with 1 mM LaCl_3_ (at 2 °C), mineralized, and the mineralization liquid was assayed for Cd^2+^ content using atomic absorption spectrophotometry (see Methods). (**c**) Kinetic of Ca^2+^ influx in maize root segments. Root segments were incubated for 15 min in the presence of 1, 5, 10, 50, 100 and 500 µM (^45^Ca^2+^)CaCl_2_. At the end of incubation, the segments were harvested, washed twice with 1 mM CaCl_2_ (non-labelled, at 2 °C), and radioactivity was measured using liquid scintillation spectrometry. Data are means ± SD of three independent experiments run in triplicate (*n* = 3).

One complication which arises in studying cation transport in walled cells (bacteria, fungi and plants) is the presence of a cell wall surrounding the plasma membrane. In the plant cell wall compartment, ions can be free in the wall pores or electrostatically bound to the negatively charged polysaccharydes and proteins that are major components of the cell wall structure [47]. In normal plant growth conditions, negative charges on the cell wall are mostly saturated by Ca ions, but this plant nutrient can be replaced by heavy metals such as Cd when exposure occurs. In fact, binding affinity of divalent, trivalent or poly cations to the cell wall has been described to be particularly high [48,49]. To address these issues, we initially set up control experiments in which root segments were incubated in 50 µM Cd^2+^ for increasing lengths of time and then washed twice in cold water (2 °C) or in a cold solution (2 °C) containing LaCl_3_, (Figure 1a, filled and open triangles, respectively). When root segments were washed with water and then harvested for Cd^2+^ analysis, the rate of Cd^2+^ accumulation was high in the first 20 min and then decreased, becoming linear (Figure 1a, filled triangles). We could fit this time-course of Cd^2+^ accumulation with a non-saturating curve that could be properly described by a modified hyperbolic function—consisting in a saturable and a linear component—of the form:

(1) $$y=\frac{ax}{b+x}+cx$$

where *y* is the Cd content in the root segments, *x* is the time and *a*, *b* and *c* are the parameters of best fitting (R = 0.999). The rate of Cd^2+^ uptake, *c*, calculated from the linear component was 2.38 ± 0.09 µmol h^−1^ g^−1^ FW.

We interpreted the saturable component as Cd ions initially diffusing rapidly into the root segments’ free space and binding to the non-diffusible anions of the cell wall, and the linear component as Cd ions steadily entering the symplast after saturation of the extracellular compartments. This interpretation was validated by washing the root segments at the end of Cd^2+^ incubation with 1 mM with La^3+^ (twice at 2 °C) to exchange the Cd ions non-absorbed by the cells and retained in the root apoplast. The results are shown in Figure 1a, open triangles. Time-depended Cd^2+^ accumulation in the root segments appeared almost linear for at least 45 min (R = 0.996), and we defined the slope of the fitted straight line as the initial Cd^2+^ influx into the symplast. The rate of Cd^2+^ uptake was 2.21 ± 0.07 µmol h^−1^ g^−1^ FW, a value very close to that calculated on the base of the linear component observed in root segments washed only with water.

Figure 1b reports the kinetic of Cd^2+^ influx as a function of Cd^2+^ external concentrations in the range of 0 to 500 µM. The influx of Cd^2+^ increased markedly up to an external concentration of 50 µM; at higher concentrations, the influx increased slowly without reaching saturation. Data analysis revealed that the kinetic profile of Cd^2+^ influx could be best described (R = 0.999) by a function sum of a saturable rectangular hyperbola and a straight line, as follows:

(2) $$v=\frac{V_{max}S}{\left(S+K_{m}\right)}+kS$$

where *v* is the rate of Cd^2+^ influx, *S* is Cd^2+^ concentration, *V_max_* and *K_m_* are the Michaelis–Menten constants and *k* is the first-order rate coefficient. The apparent *K_m_* was 30.15 ± 3.08 µM, while the *V_max_* was 3.01 ± 0.16 µmol h^−1^ g^−1^ FW; the first-order rate coefficient (*k*) of the linear component was 0.0013 ± 0.0003 L h^−1^ g^−1^ FW. To verify that the linear component of Cd^2+^ influx was not an artefact of incomplete desorption of Cd ions from the apoplast at cadmium concentrations higher than 50 µM, we first disrupted the symplast by subjecting a set of root segments to three cycles of freeze-thawing and ethanol-detergent (Triton X-100) treatment; then, we used the dead root segments to run a Cd^2+^ uptake experiment following the same protocol used for the alive root segment. Cadmium concentration in the medium was 100 µM and incubation time was 30 min, followed by two washes in 1 mM LaCl_3_. Lanthanum washing removed more than 97% of Cd^2+^ from the dead root segments (data not shown), indicating that the linear component observed in the kinetic of Cd^2+^ uptake on alive root segments (Figure 1b) was due to Cd^2+^ influx into the cells, and probably to an integral part of the complex uptake mechanism of this heavy metal by the roots.

### 2.2. Short Term Ca^2+^ Influx in Maize Root Segments

Calcium is a well-known plant macronutrient which plays critical structural and functional roles in the plant cell, including metabolism and signal transduction [50,51,52,53]. Since Cd^2+^ has been shown to interfere with Ca^2+^ functions [10], we thought to compare the kinetic of Cd^2+^ influx into root segments to that of Ca^2+^ in the same material, to highlight possible differences in the kinetic profiles of two chemically similar divalent cations, a plant nutrient and a non-essential metal. Figure 1c shows the kinetic of Ca^2+^ uptake determined in a wide range of external concentrations (from 1 to 500 µM). In these experiments, radioactive ^45^Ca^2+^ was used as a tracer, and the washes to remove labelled ^45^Ca^2+^ from the apoplast were performed using non-labelled CaCl_2_ (see Methods). Unlike the kinetic of Cd^2+^ influx, Ca^2+^ influx isotherm could be described by a simple Michaelis–Menten function (R = 0.999), with an apparent *K_m_* value of 26.57 ± 0.80 µM and a *V_max_* value of 1.24 ± 0.01 µmol h^−1^ g^−1^ FW (Figure 1c).

### 2.3. Effect of Ca^2+^ on Cd^2+^ Influx in Maize Root Segments

The kinetic analysis was extended considering the competitions between Cd^2+^ and Ca^2+^. As shown in Table 1, the presence of Ca^2+^ in the incubation medium reduced Cd^2+^ influx into the root segments. Such an inhibition closely depended on the Ca^2+^/Cd^2+^ ratio in the incubation medium. For instance, at 10 µM Cd^2+^ in the external solution an equimolar amount of Ca^2+^ reduced Cd^2+^ influx by 18%; Cd^2+^ influx was further inhibited by 58% or 70% in the presence of 50 or 100 µM Ca^2+^ in the external medium, respectively. Similar trends of inhibition were observed when Cd^2+^ influxes at 50 or 100 µM Cd^2+^ were measured in the presence of 10, 50 or 100 µM Ca^2+^.

### 2.4. Cd^2+^ and Ca^2+^ Efflux from Subapical Maize Root Segments

Pulse-chase experiments were performed to evaluate possible Cd^2+^ efflux across the plasma membrane of the cells forming the maize root segments. These experiments require loading of the root segments with radioactive Cd^2+^ for a short period of time (pulse), and measurements of Cd^2+^ leaked from the segments over longer time periods (chase). We took the precaution of using concentrations of Cd^2+^ as low as possible to avoid toxicity and long-term accumulation of chelating agents that could reduce the activity of free Cd ions in the root cells [26,27,32]. In detail, root segments were pre-loaded (15 min) in a basal medium containing 10 μM (^109^Cd^2+^)Cd^2+^ at the specific activity of 1.68 kBq nmol^−1^ (pulse phase), quickly washed in a Cd-free basal medium (washing phase, 3 × 1 min) and incubated in the same basal medium supplied with unlabelled 10 μM Cd^2+^ (chase phase). The washing phase removed radioactivity from the external surface of the segments but not Cd ions present in the free space (cell walls and intracellular spaces), as already shown in Figure 1a. Since the amount of Cd ions present in the root segments before Cd^2+^ exposure was negligible (0.18 ± 0.04 nmol g^−1^ FW), we can reasonably assume that at the end of the washes, the specific activity of (^109^Cd^2+^)Cd^2+^ into the root segments was the same as that administered during the pulse phase (1.68 kBq nmol^−1^).

During the short pulse phase, the root segments took up (^109^Cd^2+^)Cd^2+^. After this phase and the wash phase, we could estimate the amount of Cd ions accumulated in the segments by using the following parameters: (i) the decrease in radioactivity present in the medium (*A*); (ii) the radioactivity lost during the three Cd^2+^ free washings (*B*, *C*, *D*); and (iii) the specific activity of (^109^Cd^2+^)Cd^2+^ (*E*), to solve the equation below:

(3) $$Cd^{2+}accumulated=\frac{A-\left(B+C+D\right)}{E}=0.277\pm 0.001\;mol\;g^{-1}FW$$

Such a value includes Cd ions accumulated in the symplast and the free spaces of the root segments and corresponds to a total radioactivity of 465 kBq g^−1^ FW.

**Figure 2:**
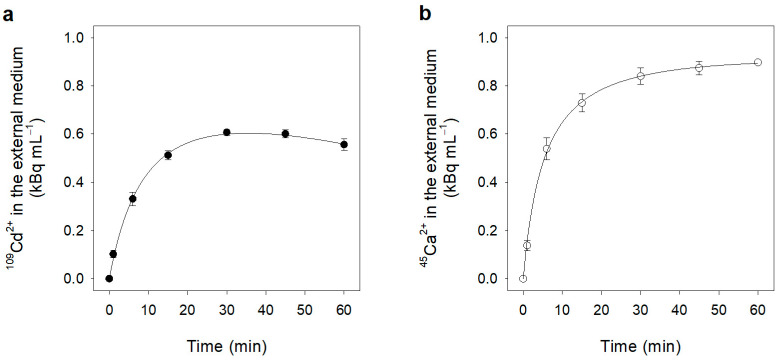
(**a**) Changes in the levels of ^109^Cd^2+^ in the external medium during the chase phase in pulse-chase experiments. (**b**) Changes in the levels of ^45^Ca^2+^ in the external medium during the chase phase in pulse-chase experiments. Data are means ± SD of three independent experiments run in triplicate (*n* = 3).

Figure 2 shows the time course of (^109^Cd^2+^)Cd^2+^ leakage from the root segments measured during the chase. Data distribution revealed that two distinct phases characterized the chase. In the first phase (0–30 min) radioactivity was rapidly released in the medium, while in the second phase (30–60 min) the radioactivity released in the medium was partially reabsorbed by the root segments. The radioactivity released in the first 30 min corresponded to 55 kBq g^−1^ FW, which is less than 12% of the (^109^Cd^2+^)Cd^2+^ present in the root segments (symplast + free space). The overall trajectory of the chase curve was interpreted as the sum of a rectangular hyperbola and a straight line (R = 0.999) as follows:

(4) $$kBq\;mL^{-1}=\frac{at}{\left(b+t\right)}+ct$$

where *t* is time, *a* (1.03 ± 0.08 kBq mL^−1^) is the theoretical maximum radioactivity at saturation, *b* (10.95 ± 1.49 min) is the *t*_1/2_ of the saturable component and *c* (−0.005 ± 0.001 kBq min^−1^ mL^−1^) is the first-order rate coefficient.

Since the apparent (^109^Cd^2+^)Cd^2+^ leakage approached saturation after 30 min, we can reasonably suppose that such an event was mainly related to the equilibration of (^109^Cd^2+^)Cd^2+^ in the root free space with the unlabelled Cd ions in the basal medium. Conversely, the second phase, in which the radioactivity of the medium linearly decreased, mainly depended on (^109^Cd^2+^)Cd^2+^ re-absorption.

Considering the first-order rate coefficient, *c*, of the linear component of the fitting equation and the specific activity of (^109^Cd^2+^)Cd^2+^ measured after 30 min from the beginning of the chase (0.061 kBq nmol^−1^), we can calculate that during the second phase of the chase, the net uptake (influx minus efflux) of Cd^2+^ into the root segments was 0.52 ± 0.09 μmol h^−1^ g^−1^ FW. Since this value is close to that of Cd^2+^ influx (0.752 ± 0.040 μmol h^−1^ g^−1^ FW), measured at the same Cd^2+^ external concentration (10 μM; Figure 1b), we can reasonably consider Cd efflux as negligible.

Similar pulse-chase experiments were carried out using radioactive Ca^2+^ as a tracer (10 μM (^45^Ca^2+^)Ca^2+^ at the specific activity of 1.72 kBq nmol^−1^). After a pulse phase of 15 min and three washes in a Ca^2+^ free medium, the total radioactivity present in the root segments was 272 kBq g^−1^ FW. In these experiments, however, we were not able to impose Ca^2+^ specific activities in the different cell compartments and in the free space, since Ca ions were initially present in the root segments as natural costituents. Figure 2b shows that during the first 30 min of the chase phase, the radioactivity rapidly increased in the external medium and then remained almost constant up to the end of the experiment (60 min). The radiocatrivity released in the medium after 30 min (80 kBq g^−1^ FW) was 29.3% of the total radioactivity present in the root segments at the beginning of the chase phase. Such a value not only depends on the rapid equilibration of (^45^Ca^2+^)Ca^2+^ in the root free space with the unlabelled Ca ions in the external medium, but also on the equilibration of (^45^Ca^2+^)Ca^2+^ present in other cell compartments. Taken as a whole, these data suggest that Ca^2+^ efflux is significantly higher than Cd^2+^ efflux.

### 2.5. Cd^2+^ Transport in Plasma Membrane Vesicles from Maize Root Cortical Cells

To validate the results obtained from intact root, Cd^2+^ transport was studied on plasma membrane preparations. Plasma membrane vesicles were purified from maize root cortical cells using a sorbitol-dextran discontinuous gradient, which produced mainly right-side-out vesicles that cannot be energized with ATP in the assay medium. To address this experimental inconvenience, three freeze-thawing cycles were applied to the vesicles, producing a mixture of inside-out and right-site-out vesicles. Only the inside-out vesicles can use ATP to energize transport, as the cytoplasmic side of the vesicle membrane is accessible to ATP in the external medium [54].

**Figure 3:**
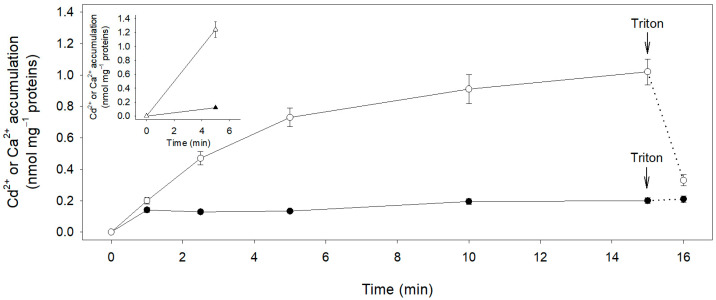
Time course of Cd^2+^ and Ca^2+^ accumulation in plasma membrane vesicles isolated from maize root cortical cells. Cd^2+^ (filled circles) and Ca^2+^ (open circles) accumulation in the vesicles were measured in the presence of protonophores. Arrows indicate the addition of Triton X-100 to the incubation media. The inset shows the proton dependent accumulation of Cd^2+^ (filled triangles) and Ca^2+^ (open triangles) in the vesicles measured in the absence of protonophores. Data are means ± SD of three independent experiments run in triplicate (*n* = 3).

In a first set of experiments we included protonophores (CCCP and ammonium) in the assay medium to exclude ion transport events dependent on the proton electrochemical gradient across the membrane vesicles generated by the activity of the Plasma Membrane H^+^-ATPase [55]. Figure 3 shows the time course of Cd^2+^ and Ca^2+^ accumulation into inside-out vesicles; such activities correspond to ion effluxes from the cells (Supplementary Figure S1). (^109^Cd^2+^)Cd^2+^ reached its maximum accumulation value in the vesicles in just the first minute of incubation, so its concentration in the vesicle was practically independent of the incubation time. The addition of Triton-X100 at the end of the incubation period did not significantly affect the level of radioactivity recovered from the vesicles. On the other hand, (^45^Ca^2+^)Ca^2+^ was taken up by the vesicles, and its concentration progressively increased as a function of time. When Triton X-100 was added to the incubation medium, Ca^2+^ level in the vesicles decreased, reaching a value similar to that measured for Cd^2+^. Such a finding strongly suggests that most of the divalent cations recovered from the vesicles after 1 min incubation were firmly bound to the membranes. Finally, since the amount of Cd ions accumulated in the vesicles never exceeds the amount of Cd ions bound to the membranes, we can conclude that Cd^2+^ efflux through the vesicle membranes is negligible. In contrast, the ATP-dependent (^45^Ca^2+^)Ca^2+^ accumulation in the vesicles underlines that Ca^2+^ efflux is an activity of the plasma membrane of the root cortical cells (Supplementary Figure S1). The presence of protonophores did not affect the accumulation of (^109^Cd^2+^)Cd^2+^ in the plama membrane vesicles after 5 min incubation, as the levels of (^109^Cd^2+^)Cd^2+^ were similar in the presence or absence of protonophores (Figure 3, inset). On the other hand, (^45^Ca^2+^)Ca^2+^ was 50% higher in the absence of protonophores, indicating that the transport activities of Ca^2+^ closely dependent on the proton electrochemical gradient cooperate with Ca^2+^-ATPases to facilitate Ca^2+^ efflux from root cortical cells (Supplementary Figure S1) [56,57,58].

## 3. Discussion

Results of the initial experiments shown in Figure 1a indicate that, following La^3+^ washing, Cd^2+^ accumulation into the root segments was linear for at least 45 min. In addition, since only traces of Cd^2+^ were present in the root segments (0.18 ± 0.04 nmol g^−1^ FW) before cadmium exposure, we can reasonably consider Cd^2+^ accumulation as the result of a unidirectional flux (influx). Such a finding allowed us to determine the kinetic isotherm of Cd^2+^ uptake under a wide range of Cd^2+^ external concentration (Figure 1b).

The kinetic analysis showed that the pattern of Cd^2+^ influx into root cells followed a complex isotherm, which was a result of the combination of a saturable and a first-order linear kinetic. This suggests that multiple transporters for essential elements with insufficient selectivity with respect to their substrate, such as Ca^2+^ channels, LCT1, ZIP-IRT and NRAMP transporters [11,14,14,38,59,60,61], were working in parallel at the plasma membrane to allow Cd^2+^ absorption into root cells. The apparent *K_m_* (30.15 ± 3.08 μM) of the saturable component of the kinetic was higher compared to the value measured in intact rice roots [14]; such a discrepancy may be due to the different species and/or organs used for the experiments. By contrast, kinetic of Ca^2+^ influx in the root segments (Figure 1c) could be described by a simple Michaelis–Menten function with an apparent *K_m_* value of 26.57 ± 0.80 μM. Such a value could be underestimated since it was calculated using the administered specific activity of (^45^Ca^2+^)Ca^2+^ in the medium without considering the Ca^2+^ present in the tissues. Nevertheless, a direct comparison of the *K_m_* values measured for Cd^2+^ and Ca^2+^ influxes does not allow us to exclude the hypothesis that the two ions may partially share the same transport system(s), as previously observed in wheat [38]. Moreover, in our experimental setup the presence of Ca^2+^ in the incubation medium significantly reduced Cd^2+^ influx in the root segments (Table 1), suggesting the existence of a competition between the two ions for the same transport system(s). Cd^2+^/Ca^2+^ competitions have also been suggested following the observation that Ca ions may alleviate Cd^2+^ toxicity in different plant species [22,62,63].

Cadmium concentration in the cells could be reduced by the activity of transporters mediating Cd^2+^ efflux across the plasma membrane [64,65,66]. Similar mechanisms have been largely described as involved in controlling Na^+^ [67] and Ca^2+^ [56] homeostasis. Mechanisms controlling Ca^2+^ concentration in the cells are essential to prevent potentially toxic effects due to the aggregation of proteins, nucleic acids and phosphates, as well as to allow the role of this ion as a second messenger [50,51,68].

A possible activity mediating Cd^2+^ efflux from the root segments has been investigated here using a pulse-chase approach with (^109^Cd^2+^)Cd^2+^ (Figure 2a). Notwithstanding the presence of a root free space limiting the possibility of performing a precise measurement of Cd^2+^ efflux, results clearly indicate that when this compartment equilibrated with the external medium (after 30 min from the beginning of the chase phase), an apparent Cd^2+^ re-influx occurred (30–60 min of the chase phase). Since the rate of Cd^2+^ net uptake measured during this last period was similar to that of Cd^2+^ influx measured at the same external concentration (Figure 1b), we can conclude that, in our experimental conditions, Cd^2+^ efflux from the root segments is minimal. On the other hand, results of similar experiments carried out with (^45^Ca^2+^)Ca^2+^ (Figure 2b) indicate that after the equilibration of the root free space with the external medium, ^45^Ca^2+^ was not re-absorbed by the segments, probably as the result of an equilibrium between Ca^2+^ influx and efflux.

The analysis of Cd^2+^ fluxes measured in inside-out vesicles also indicated the absence of a Cd^2+^ efflux activity on the plasma membrane of the root cortical cells both in the presence or in the absence of protonophores (Figure 3, Supplementary Figure S1). On the contrary, the energization of the vesicles with ATP resulted in significant Ca^2+^ effluxes in the presence or absence of protonophores (Figure 3, Supplementary Figure S1), suggesting the existence of both ATP- and proton-dependent activities mediating Ca^2+^ efflux on the plasma membrane of the root cortical cells [56,57,58].

Taken as a whole, our results indicate that Cd^2+^ influx into the root cells is mediated by a complex mechanism that involves different plasma membrane transport proteins, including Ca^2+^ transporters. Moreover, the cortical cells of the maize root are not able to extrude Cd^2+^ and show at the same time a highly selective activity of Ca^2+^ extrusion, as already suggested by Rivetta and co-workers [22].

From an evolutionary viewpoint, our data could support the heuristic hypothesis that the impossibility of extruding a large amount of Cd^2+^ from the root cortical cells could have imposed strong selective pressure to favour the evolution of protein or non-protein peptides (i.e., metallothioneins and phytochelatins) [69] able to bind and detoxify Cd^2+^ inside the cells.

## 4. Materials and Methods

### 4.1. Plant Material

Maize (*Zea mays* L., cv. Dekalb 300) caryopses were germinated in the dark at 26 °C on filter paper saturated with milli-Q water. After 48 h, seedlings were transferred to aerated 0.5 mM CaSO_4_ solutions and maintained in the dark at 26 °C for 24 h; at the end of this period, the main root was about 40–50 mm long. Subapical root segments (6 mm long) were obtained by cutting the main root at 2 and 8 mm from the tip. Batches of 20 segments (about 100 mg of fresh weight) were then washed twice for 30 min in 10 mL of a 0.1 mM Mes-BTP (pH = 6.0) basal medium, and incubated in a water bath thermoregulated at 26°C and reciprocated at 90 oscillations min^−1^; after these treatments (preincubation), the segments had completely recovered from wounding.

### 4.2. Measurement of Cd^2+^ Influx

After preincubation, subapical root segments were incubated at different times in 10 mL of basal medium supplemented with CdCl_2_ at different concentrations at 26 °C and agitated at 90 oscillations min^−1^. At the end of the incubation period, the segments were washed twice at 2 °C for 15 min in milli-Q water or in the basal medium supplemented with 1 mM LaCl_3_ and then mineralized at 100 °C in 0.5 mL of HNO_3_-H_2_SO_4_-HClO_4_ (5:1:1, v:v:v). The amount of Cd^2+^ in the root segments was determined by atomic absorption spectrophotometry (SpectraAA-20, Varian, Palo Alto, CA, USA). The effect of Ca^2+^ on Cd^2+^ influx was measured by incubating the root segments in solutions containing 10, 50 or 100 µM Cd^2+^ in the presence of variable Ca^2+^ concentrations (from 0 to 100 µM). Experiments of Cd^2+^ uptake were also carried out using segments three times frozen-thawed and then washed four times (30 min each) in absolute ethanol containing 2% (*w*:*v*) Triton X-100 (Sigma-Aldrich, Saint Louis, MI, USA) and finally extensively rinsed in the basal medium.

### 4.3. Measurement of Ca^2+^ Influx

Twenty preincubated root segments were incubated at 26 °C for 15 min in 10 mL of basal medium supplemented with CaCl_2_ at different concentrations and agitated at 90 oscillations min^−1^; solutions were labelled with 3.7 kBq mL^−1 45^Ca^2+^. At the end of the incubation period, the segments were washed twice at 2 °C for 15 min in the basal medium supplemented with 1 mM CaCl_2_ and then rinsed in milli-Q water. The segments were then homogenized in 0.75 mL of 0.1 N HNO_3_ and heated at 100 °C for 15 min. Radioactivity was measured by counting, in a Beckman LS 6000S (Fullerton, CA, USA) scintillation counter, aliquots of the supernatants dissolved in 10 mL of Ready-Solve scintillation cocktail (Backman).

### 4.4. Pulse and Chase Experiments with (^109^Cd^2+^)Cd^2+^ or (^45^Ca^2+^)Ca^2+^

Cd^2+^ and Ca^2+^ fluxes were measured using 10 subapical maize root segments incubated at 26 °C for 15 min in 5 mL of basal medium containing 10 μM (^109^Cd^2+^)CdCl_2_ or 10 μM (^45^Ca^2+^)CaCl_2_, at the specific activity of 1.68 kBq nmol^−1^ or 1.72 kBq nmol^−1^, respectively. At the end of the pulse phase, the root segments were washed three times for 1 min in 5 mL of Mes-BTP (pH = 6) and then incubated at 26 °C for 60 min in 5 mL of basal medium supplemented with 10 μM non-radioactive Cd^2+^ or Ca^2+^ (chase phase). Radioactivity was measured in all the liquid media (at the end of the pulse phase, at the end of the washes and at different time during the chase phase) by liquid scintillation spectrometry.

### 4.5. Plasma Membrane Vesicle Isolation

Plasma membrane vesicles were prepared from maize root cortex using a method based on the one described by Palmgren and co-workers [54]. The cortex was separated from the stele, using a wire stripper, and blended with a solution of 330 mM sorbitol, 50 mM Mops-BTP (pH = 7.5), 5 mM EDTA, 1 mM DTT, 0.5 mM PMSF and 1% (*w*:*v*) BSA. The resulting mixture was filtered and centrifuged at 13,000× *g* for 15 min, and the supernatant was subjected to further high-speed centrifugation at 100,000× *g* for 30 min to obtain the microsomal fraction. This fraction was then suspended in a solution of 330 mM sorbitol, 5 mM KCl, 1 mM DTT, 0.1 mM EDTA and 5 mM K-phosphate (pH = 7.8). The plasma membrane vesicles were purified using a three-step sorbitol/dextran T-500/PEG 3350 gradient [70]. The product was suspended in a solution of 330 mM sorbitol, 50 mM KCl and 2 mM Mops-BTP (pH = 7.0) to obtain a final protein concentration of about 8 µg/µL. The plasma membranes were then frozen-thawed three times to obtain a mixture of inside-out and right-side-out vesicles [54].

### 4.6. Measurement of Ca^2+^ and Cd^2+^ Uptake in Plasma Membrane Vesicles

The uptake of Ca^2+^ and Cd^2+^ was measured in plasma membrane vesicles using ^45^Ca^2+^ and ^109^Cd^2+^. The vesicles (100 µg protein sample^−1^) were pre-incubated for 60 min at 30 °C in a solution containing 0.6 mL of 250 mM sorbitol, 25 mM Tris-Mes (pH = 7.2), 1 mM ATP, 0.015% (*w*:*v*) Brij 58, and 10 µM (^45^Ca^2+^)CaCl_2_ (1 kBq ^45^Ca^2+^ mL^−1^) or 10 µM (^109^Cd^2+^)CdCl_2_ (1 kBq ^109^Cd^2+^ mL^−1^), in the presence or absence of 100 µM CCCP, 5 mM (NH_4_)_2_SO_4_. The uptake was initiated by adding MgSO_4_ to reach a final concentration of 1 mM. At various times, samples were filtered through a 0.22 µm filter (GSWP, Millipore Corp, Burlington, MA, USA) and rapidly washed with 4 mL of a solution of 25 mM Tris-Mes (pH = 7.2) containing 1 mM CaCl_2_ or 1 mM CdCl_2_. Filters were dried at 45 °C for 30 min, and radioactivity was measured by liquid scintillation spectrometry. MgATP-dependent uptake of Ca^2+^ or Cd^2+^ was determined by subtracting the radioactivity measured in the absence of Mg^2+^ from that measured in its presence.

### 4.7. Statistical Analysis

The results presented are the average of three experiments, each conduced in triplicate (*n* = 3). Statistical analysis was performed using SigmaPlot for Windows version 11.0 (Systat Software, Inc., Point Richmond, CA, USA), and the significance of the results was determined using ANOVA. A *p* value of less than 0.05 was considered significant. The Bonferroni correction was applied to account for multiple comparisons. The fitting of the data was performed using SigmaPlot for Windows version 11.0.

## Figures and Tables

**Table 1:** Effect of Ca^2+^ on Cd^2+^ influx in subapical maize root segments.

[Ca^2+^] (µM)	[Cd^2+^]					
	10 µM		50 µM		100 µM	
	Cd^2+^ influx (µmol h^−1^ g^−1^ FW)	Δ (%)	Cd^2+^ influx (µmol h^−1^ g^−1^ FW)	Δ (%)	Cd^2+^ influx (µmol h^−1^ g^−1^ FW)	Δ (%)
0	0.79 ± 0.04 a		1.97 ± 0.03 a		2.32 ± 0.12 a	
10	0.65 ± 0.03 b	−17.7	1.68 ± 0.02 b	−14.7	2.19 ± 0.08 a	−5.6
50	0.33 ± 0.02 c	−58.2	1.32 ± 0.09 c	−33.0	1.72 ± 0.04 b	−25.9
100	0.24 ± 0.01 d	−69.6	1.11 ± 0.02 d	−43.7	1.47 ± 0.04 c	−36.6

Maize root segments were incubated for 15 min in solutions containing 10, 50 or 100 µM CdCl_2_ in the presence of variable CaCl_2_ concentrations (from 0 to 100 µM). At the end of the incubation, root segments were washed twice in 1 mM LaCl_3_. Cadmium in the root segments was assayed as indicated in Figure 1b. Data are means *±* SD of three independent experiments run in triplicate (*n* = 3). Different letters within a data column indicate significant differences between calcium treatments (*p* < 0.05).

## Data Availability

The raw data supporting the conclusions of this article will be made available by the authors, without undue reservation, to any qualified researcher.

## Supplementary Materials

The following supporting information can be downloaded at: https://www.mdpi.com/article/10.3390/plants12050992/s1, Figure S1: Ca^2+^ accumulation in inside-out plasma membrane vesicles in the presence (a) or absence (b) of protonophores. Cd^2+^ accumulation in inside-out plasma membrane vesicles in the presence (c) or absence (d) of protonophores.
