# Supplementary material for: Cadmium Transport in Maize Root Segments Using a Classical Physiological Approach: Evidence of Influx Largely Exceeding Efflux in Subapical Regions
Source: Plants (Basel). 2023 Feb 21;12(5):992. doi: 10.3390/plants12050992 (PMC10005225; doi:10.3390/plants12050992)

Supplementary Figure S1. Ca^{2+} accumulation in inside-out plasma membrane vesicles in the presence (**a**) or absence (**b**) of protonophores. Cd^{2+} accumulation in inside-out plasma membrane vesicles in the presence (**c**) or absence (**d)** of protonophores. Ion accumulations in the vesicles correspond to ion effluxes from the cells. The electrochemical proton gradient across the plasma membrane is established and maintained by plasma membrane H^+-ATPases (Panel **b**). In this case, Ca^{2+} accumulation in the vesicles is mediated by ATP-dependent (Ca^{2+}-ATPases) and H^+- dependent (CAX?) Ca^{2+} transporters. Protonophores dissipate the H^+ gradient, stalling the activity of H^+-dependent Ca^{2+} transporters (Panel **a**, crossed and dotted arrow line) allowing Ca^{2+} movement only through Ca^{2+}-ATPases. When cadmium is in the external medium, (Panels **c** and **d**), Ca^{2+} transporters are unable to move Cd^{2+} across the membrane, with or without protonophores.

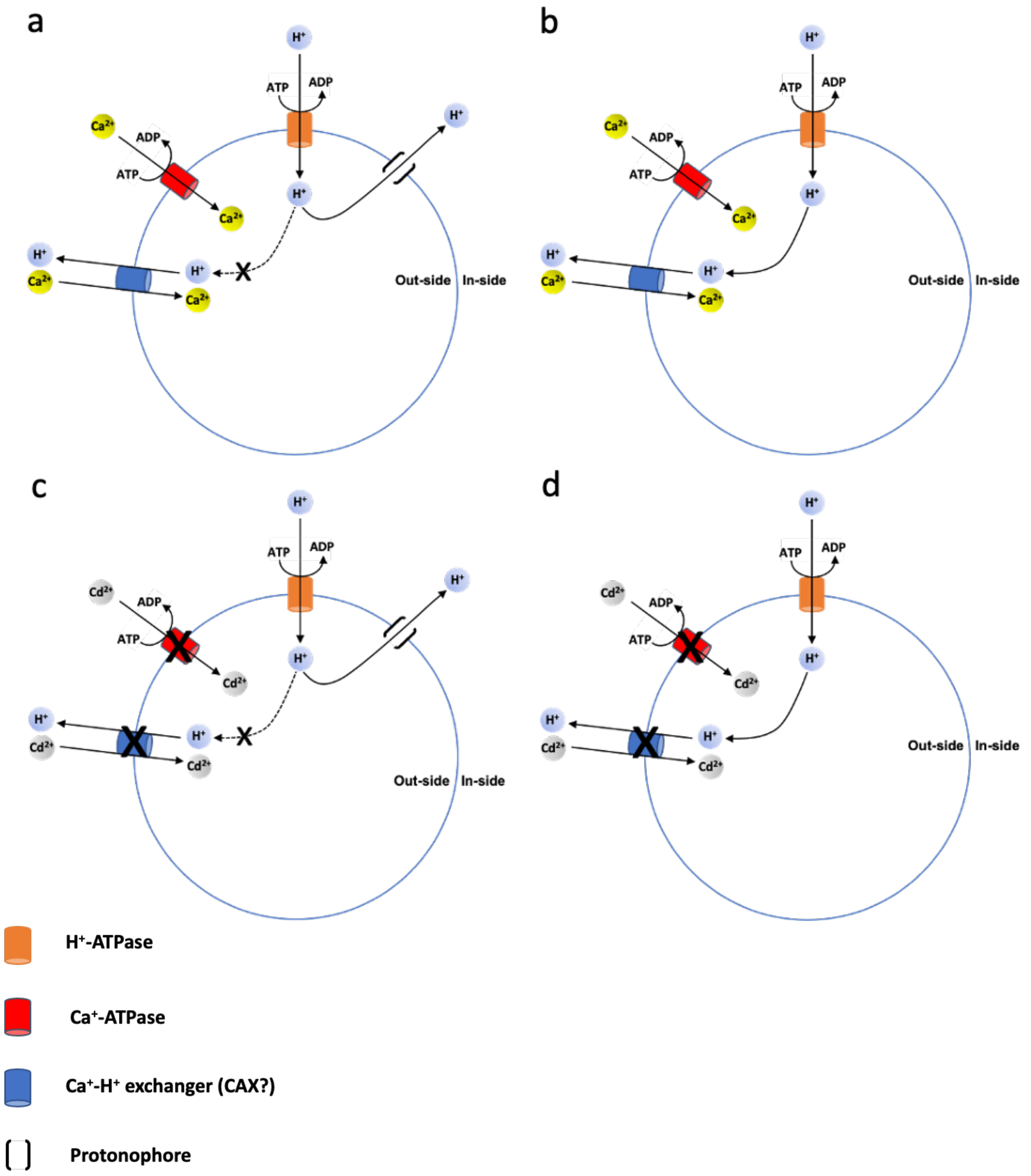

Supplement: Supplementary file 1 [file plants-12-00992-s001.zip › plants-2208032-supplementary.pdf]
